# Knowledge, attitudes, and practices of the ethics in medical research among Moroccan interns and resident physicians

**DOI:** 10.1186/s12910-024-01029-9

**Published:** 2024-03-20

**Authors:** Ibtissam El Harch, Soumaya Benmaamar, Nabil Tachfouti, Moustapha Hida, Mohammed Faouzi Belahsen, Tarik Sqalli Houssaini, Karima El Rhazi

**Affiliations:** 1 Epidemiology and Research in Health Sciences (ERESS) Research Laboratory, Faculty of Medicine, Pharmacy and Dentistry, Sidi Mohamed Ben Abdellah University, Fes, Morocco; 2 Pediatric department, CHU Hassan II, Fez, Morocco; 3 Neurology department, CHU Hassan II, Fez, Morocco; 4 Nephrology department, CHU Hassan II, Fez, Morocco

**Keywords:** Ethics in medical research, Knowledge, Attitudes and practices, Moroccan physicians

## Abstract

**Background:**

In Morocco, medical research ethics training was integrated into the medical curriculum during the 2015 reform. In the same year, a law on medical research ethics was enacted to protect individuals participating in medical research. These improvements, whether in the reform or in the enactment of the law, could positively impact the knowledge of these researchers and, consequently, their attitudes and practices regarding medical research ethics. The main objective of this work is to assess Moroccan physicians’ knowledge, attitudes, and practices at the beginning of their careers (interns and residents) in medical research ethics.

**Patients and methods:**

This is a multicenter cross-sectional study conducted in 2021 among Moroccan physicians. Three scores were created and validated to assess physicians’ level of knowledge, attitudes, and practices regarding research ethics. A descriptive analysis was carried out, followed by a univariate analysis and a multivariate analysis using multivariate binary logistic regression to study the factors associated with the different calculated scores.

**Results:**

A total of 924 physicians were included in the study, with an average age of 27.8 ± 2.2 years. 40.7% had a high medical research ethics knowledge score, and 68.8% had good attitudes. These two scores were positively associated with age and were statistically higher in residents and in physicians who had received training in medical research ethics during their medical curriculum. Only 29,9% of physicians who had participated in research studies had adequate practices with medical research ethics. This score was statistically higher in residents and in physicians who had heard about research ethics.

**Conclusion:**

A genuine introduction to ethics in the medical curriculum is essential to enhance researchers’ knowledge, attitudes, and practices. This, in turn, can lead to an increase in both the quantity and quality of research conducted in Morocco.

**Supplementary Information:**

The online version contains supplementary material available at 10.1186/s12910-024-01029-9.

## Introduction

Since its establishment in 1946, the World Health Organization (WHO) has defined health as « a state of complete physical, mental, and social well-being and not merely the absence of disease or infirmity » [1]. It is considered a fundamental right for everyone worldwide [2, 3]. Medical research plays a crucial role in guaranteeing this right by conducting studies on human health and disease states. These studies, whether through observation or interventions, lead to new and better recommendations for improving the health and well-being of populations.

After the Second World War, inhumane practices in medical research [4] prompted the establishment of guidelines, beginning with the Nuremberg Code in 1947 [5]. This was followed by the Declaration of Helsinki, drafted by the World Medical Association (WMA) in 1964. These guidelines contain recommendations applicable to every physician in biomedical research involving human subjects [6, 7], but despite these international guidelines prohibiting inhuman experiments [8], this was not always respected [9]. In 1974, the United States established the National Commission for the Protection of Human Subjects in Medical and Behavioral Research, leading to the creation of the Belmont Report [10]. This report outlines the four fundamental principles of medical research ethics - autonomy, justice, beneficence, and non-maleficence – and it has become a canonical document in the field [11, 12].

The Council for International Organizations of Medical Sciences (CIOMS) in collaboration with the WHO, initiated its work on the ethics of biomedical research in the late 1970s. This collaboration led to the development of guidelines aiming to provide internationally validated ethical principles and detailed commentary on how universal ethical principles should be applied [13]. These guidelines are a reference for medical research ethics [14] and should be adhered to by all researchers.

Studies have indicated that the knowledge and attitudes of medical researchers regarding medical research ethics are inadequate, particularly in developing countries [15–17]. This poses a significant challenge for medical research conducted in these countries.

In Morocco, training in medical research ethics was integrated into the medical curriculum during the 2015 reform [18]. In the same year, a law related to medical research ethics was enacted to ensure the protection of individuals participating in medical research [19]. Prior to this, Moroccan researchers relied solely on various international directives, especially the Helsinki Declaration, that were not legally binding. The integration of medical research ethics training and the promulgation of the law may positively impact the knowledge, attitudes, and practices of Moroccan researchers.

In Moroccan medical curricula, no training in medical research ethics is organized after the last year of training in general medicine. To determine whether students possess sufficient knowledge and appropriate attitudes and behaviors to conduct and/or participate in health research at the end of their medical training, we conducted this study whose main objective is to assess the knowledge, attitudes and practices, of doctors who have just completed their training in general medicine (interns and residents), in the field of medical research ethics.

## Patients and methods

### Study design

This is a multicenter cross-sectional study conducted in 2021 among Moroccan physicians from various university Hospital centers (UHC) and regional hospitals in Morocco.

### Study population

The study included periphery internal doctors (who are students in the last year of medicine and who carry out their periphery internship), interns (students who had validated all the modules and who serve a two-year internship within the UHC, which allows them to practice medicine, but only under the close supervision of professors), or resident (they are medical physicians pursuing their training in specialized departments under the close supervision of a professor). Physician not wishing to participate were excluded from this study.

Based on the official data from the UHC of Fes in September 2021, there were 820 interns and residents. Therefore, we can estimate that during the year 2021 (the year study), the five CHUs in the kingdom collectively accommodated a maximum of 4500 interns and residents.

### Questionnaire development process

The questionnaire was developed **in French** by ourselves, through a literature search and with input from a group of experts that included ethicists, medical professors, physicians, medical students, and administrative staff. The group held periodic meetings to create the initial version of the questionnaire, which includes questions on: (a) knowledge, aiming to assess participants’ information on research ethics and their understanding of the facts; (b) attitudes, encompassing questions evaluating participants’ personal perspectives, beliefs, and feelings regarding ethical considerations in research; and (c) practices, including questions assessing participants’ implementation of research ethics.

Before the final launch of the questionnaire, a pre-test was conducted at the UHC of Fes with 5 interns and 5 residents. They were asked to answer the questions, identify any non-understandable items, and suggest alternative proposals. The questionnaire was then corrected accordingly.

The knowledge section contained 19 items related to the definition of research ethics, Moroccan law governing medical research ethics, existing basic documents, fundamental principles of medical research ethics, and ethics committees.

The attitude section contained 14 items about the different attitudes adopted by physicians with respect to consent, vulnerability, confidentiality, conflicts of interest and ethics committees.

The practices section data contained 4 items about the practices of physicians who had already participated in medical research studies, with a focus on the consent procedure.

For each item, several propositions were given, and the participants were asked to choose the true ones according to their own knowledge, attitudes, and practices.

Three scores were created to assess physicians’ levels of knowledge, attitudes, and practices regarding medical research ethics. To calculate these scores, each correct answer was assigned a value of 1, while false answers were assigned a value of 0. The total score of each section was then calculated by adding the results of the corresponding items, resulting in a total score ranging from 0 to 19 for knowledge, 0 to 14 for attitudes, and 0 to 4 for practices.

The knowledge, attitudes, and practices scores were divided into 2 categories, each based on their 75th percentile: “low category” below the 75th percentile and “High category” above the 75th percentile **(The questionnaire and the score calculation guide are available in the appendix).**

### Data collection

The data was collected using an electronic questionnaire filled out directly by eligible participants, containing an initial section explaining the study’s objectives and methodology and asking participants to provide their consent by checking their agreement to participate in the study.

The target population was contacted through academic emails or social media. Upon registration at different medical faculties in the country, students receive an academic email address, which they retain permanently and use for communication with their faculties. Therefore, we obtained the email addresses of our target physicians from the different medical faculties. We also used social media, by distributing the questionnaire in the different groups of doctors and medical students, either on WhatsApp or on Facebook to maximize participant reach, as individuals tend to use social networks more frequently than their email accounts.

The questionnaire collected data on the personal characteristics of participating physicians, including age, gender, training year, and Faculty of medical education. Additionally, it gathered data on their research ethics training, distinguishing between those who received training or practical training in medical research ethics and who having heard about the ethics of medical research, meaning they have never received formal training but have some understanding of its existence, either by hearing about it in hospitals, faculties, media or by reading a scientific publication. The questionnaire also collected data on physicians’ knowledge, attitudes, and practices related to research ethics.

### Validation

After initiating data collection, we conducted a validation of the final version of the questionnaire with the initial 100 participants. The internal consistency of the 3 scores (Knowledge, Attitudes and Practices), was evaluated using Cronbach’s alpha coefficient, and considered a value of 0.70 or higher to be sufficient [20]. The construct validity was evaluated using multitrait analysis, examining correlations between items and total scores (corresponding score for convergence validity and non-corresponding scores for discriminant validity). The proof of the item’s convergence was defined as a correlation of 0.40 or more between an item and its own total score. The items discrimination was satisfied if each question had a significantly higher correlation with its total score than with the other scores [21].

All scores had good reliability (Cronbach’s alpha was 0.82 for the knowledge score, 0.86 for the practices score, and 0.88 for the attitudes score). The attitude and practice scores had 100% item convergence (r: 0.490–0.781 and r: 0.616–0.902 successively) and 100% discrimination (r: -0.247-0.550 and r: 0.077–0.435 successively). At the same time, the knowledge score had a convergence of 89.5% (17 of the 19 items exceeded the threshold of 0.40) and a discrimination of 78.9% (15 of the 19 items had a significantly higher correlation coefficient with the knowledge score compared to the other scores).

### Statistical analysis

A descriptive analysis was conducted to describe the personal data, knowledge, attitudes and practices items of all participants. Qualitative variables were reported as numbers and percentages, while quantitative variables were presented as mean ± standard deviation.

The study of the association between categories of knowledge, attitudes, and practices scores, and the different factors was carried out using simple logistic regression. The significance level was set at 5%, and the results were presented in crude Odds Ratios (ORc) along with their 95% confidence intervals.

Furthermore, a multivariate binary logistic regression analysis was performed to investigate factors associated with participants’ knowledge, attitudes, and practice scores while controlling for confounding factors (age, training year, having heard about research ethics, receiving training in research ethics while studying medicine…). The inclusion threshold for the first multivariate model was set at 20%, while 5% was set as the threshold for retaining the factors of the final model. The results were presented in adjusted ORs (Adj ORs) and their 95% confidence intervals. The statistical analysis was performed using SPSS.26 software.

## Results

The research encompassed 924 participants, leading to an estimated response rate of 20.5% (924/4500).

### General and training characteristics of participating physicians

The average age of participants was 27.8 ± 2.2 years, with a slight predominance of women (56.3%). Nearly half of the participants (48.3%) were residents. Also, 67.2% have already heard about research ethics, 10.2% had received training in medical research ethics during their medical curricula, and only 0.8% had received practical training in medical research ethics (Table 1).

**Table 1:** **General and training characteristics of participating physicians**

		*N* = 924	
		*N*	%
Age in years (m ± SD)		27.8 ± 2.2	
Gender	Women	520	56.3%
	Men	404	43.7%
Training year	Interns (1st and 2nd year) and Periphery internal doctors	478	51.7%
	Residents	446	48.3%
Faculty of medical education	Fez	262	28.4%
	Rabat	195	21.1%
	Casablanca	179	19.4%
	Oujda	138	14.9%
	Marrakech	150	16.2%
Having heard about the ethics of medical research	Yes	621	67.2%
	No or I don’t remember	303	32.8%
Having heard about the ethics of medical research in (*N* = 621)	The faculty	564	90.8%
	The hospital	305	49.1%
	Scientific publications	339	54.6%
	The media	99	15.9%
have received training in medical ethics while studying medicine	Yes	94	10.2%
	No or I don’t remember	830	89.8%
Have received practical training in medical research ethics	Yes	7	0.8%
	No or I don’t remember	917	99,2%

### Participant’s knowledge, attitudes, and practices in medical research ethics

For the evaluation of the knowledge, 50.1% knew the exact definition of research ethics, 41.8% knew that there was a Moroccan law governing research ethics, including only 5.4% who knew the exact name of that law n° 28 − 13. The basic documents on research ethics (*Nuremberg Code, Declaration of Helsinki, Belmont Report and the International Ethical Guidelines for Medical Research Involving Human Subjects*) were known by only 37.3% and 45.6% knew their fundamental principles (Table 2).

**Table 2:** Description of participant’s knowledge, attitudes, and practices in research ethics

			*N* = 924	(%)
	knowledge assessment			
Knowledge	Know the correct definition of ethics in medical research		463	50.1%
	know there is a Moroccan law governing research ethics		386	41.8%
	know that the law which governs the ethics of medical research in Morocco is law n ° 28 − 13 (*N* = 386)		21	5.4%
	know the reference documents in ethics of medical research		345	37.3%
	know the fundamental principles of ethics in medical research		421	45.6%
	know that it is mandatory to obtain informed consent from study participants		752	81.4%
	know that patients should be informed of all the potential risks of a study		616	66.7%
	know that vulnerable groups (children, mentally ill) cannot themselves give their informed consent		621	67.2%
	know that a vulnerable person cannot be included in research in the absence of their legal representative who must give informed consent on their behalf		621	67.2%
	know that the use of anonymous data is a means of protecting the privacy and confidentiality of research participants		669	72.4%
	know that informed consent includes the right of subjects to withdraw him/herself from the study at any time		701	75.9%
	know that the risks and benefits of research should be shared equally among study participants		732	79.2%
	know that researchers can not in any way exclude people from participating in a research project because of their characteristics (culture, language, etc.)		596	64.5%
	know that researchers must specify the reasons of exclusions of certain groups from their research project to their ethics committees		638	69%
	know that confidentiality is broken if a researcher discloses information that puts the participant at risk of injury/harm / illness		777	84.1%
	know that a person lacking autonomy should be reassessed regularly		665	72%
	know that there are ethics committees in Morocco		501	54.2%
	know that there is an ethics committee within the faculty		530	57.4%
	know the functions attributable to the medical research ethics committee		435	47.1%
	Knowledge score	High	376	40.7%
		Low	548	59.3%
	Physicians, in case of participating in a research study, should:			
Attitudes	always ask for permission or inform the participant before the his/her inclusion in the study		775	83.9%
	Maintain the confidentiality of participant information to the extent possible, except in situations where there is a risk of harm to others		760	82.3%
	Keep the informed consent forms locked and separate from research files to protect the privacy and confidentiality of the participant		734	79.4%
	Explain informed consent to the participant in their local language		765	82.8%
	inform the research participants if they will be compensated in the event of injury due to the protocol		666	72.1%
	Inform the participants of complete information on the research protocol (duration, risks, etc.)		716	77.5%
	Require an informed consent when using their biological samples in research		644	69.7%
	Not conducting research on a vulnerable subject when it can be conducted on a normal subject.		686	74.2%
	Not seeking the decision to participate in research from vulnerable subjects		643	69.6%
	Requesting the mandatory presence of a legal representative during the informed consent process for vulnerable persons		680	73.6%
	Not recommending the participation of patients in medical research when under financial, administrative, hierarchical or political pressure.		655	70.9%
	Not be agree to inappropriately alter or suppress the results of research as a result of financial, administrative, hierarchical or political pressure or inducement		686	74.2%
	Inform participants in medical research of the existence of any conflict of interest and how it is managed.		596	64.5%
	Agree to be supervised by ethics committees		824	89.2%
	Attitudes score	Adequate attitudes	636	68.8%
		Inadequate attitudes	288	31.2%
	Practices in research ethics among participants who have already participated in medical research (*N* = 119)			
Practices	Have requested informed consent during my researches		96	80.7%
	Have already explained to the participants that they are taking part in a research-based study		87	73.1%
	Requested informed consent from participants in their local language		86	72.3%)
	Have requested informed consent from the guardian or legal representative of a vulnerable participant (*N* = 117)		32	27.4%)
	Practices score (*N* = 117)	Adequate practices	29.4	29.9%
		Inadequate practices	68.9	70.1%

The 75th percentile for the knowledge score was 14.5, indicating that 40.7% of participants had a high knowledge score. This score was positively associated with age and statistically higher among physicians who had already heard of or received training in medical research ethics and among residents (Table 3).

**Table 3:** Associated factors with knowledge, attitudes, and practices scores

		knowledge score		Attitudes score		Practices score	
		OR_c_ (CI)	OR_Adj_ (CI)	OR_c_ (CI)	OR_Adj_ (CI)	OR_c_ (CI)	OR_Adj_ (CI)
Age		1.4 (1.3–1.5)	1.2 (1.1–1.4)	1.4 (1.3–1.5)	1.2 (1.1–1.4)	-	-
Training year	Intern AND Periphery internal doctors	1	1	1	1	1	1
	Resident	4.0 (3.0–5.3)	2.1 (1.4–3.3)	3.4 (2.5–4.7)	1.8 (1.1–3.0)	4.9 (2.1–11.7)	3.6 (1.5–8.9)
Having heard about research ethics	No or I don’t remember	1	-	1	-	1	1
	Yes	47.9 (23,9–93,4)	-	12.1 (8.7– 16.8)	-	5.4 (2.1–14.2)	3.4 (1.2–9.5)
have received training in research ethics while studying medicine	No or I don’t remember	1	1	1	1	-	-
	Yes	2,0 (1.3–3.1)	2.4 (1.5–3.8)	2.0 (1.7–5.6)	3.7 (1.9–7.1)	-	-
Knowledge score	Low	-	-	-	-	1	-
	High	-	-	-	-	2.6 (1–6.6)	-

Regarding attitudes, physicians showed good attitudes towards research ethics regarding consent, confidentiality, ethics committees, vulnerable people, and conflicts of interest.

The 75th percentile of this score was 10.5, indicating that 68.8% of physicians had good attitudes towards medical research ethics (Table 2**)**. This score was statistically higher among older physicians, those who had already heard of or received training in medical research ethics, and among residents (Table 3).

Regarding practices, only 12.9% of physicians (*N* = 119) had participated in health research studies, of which 80.7% obtained informed consent from their patients. The 75th percentile of this score was 3, indicating that only 29.9% of physicians who had participated in research studies had adequate practices with respect to medical research ethics (Table 2**)**. This score was statistically higher among physicians who had heard of research ethics, residents, and those with high knowledge scores (Table 3).

Multivariate analysis (Table 3**)** showed that, on the one hand, older age, physicians who had received research ethics training during their medical curriculum, and residents had statistically the highest knowledge and attitude scores, whereas having heard about research ethics was not associated with these scores. On the other hand, physicians who had heard about research ethics and residents had statistically highest practices score, while age, receipt of research ethics training during their medical curriculum, and knowledge score were not associated with this score.

## Discussion

The main objective of this work was to assess the level of knowledge, attitudes and practices of Moroccan interns and residents regarding medical research ethics. It was found that 40.7% had a high knowledge score and 68.8% had adequate attitudes. These two scores were associated with advanced age and were significantly higher among residents and physicians who had already received training in research ethics during their medical curriculum. For the practices score, only 12.9% of the physicians had ever participated in a research study, and 29.9% of them had adequate practices.

The knowledge of the definition of medical research ethics, its fundamental principles, and its main guidelines were weak, which is the case in most developing countries [17, 22]. Additionally, 41.8% of the physicians did not know that there is a Moroccan law governing medical research, and only 5.4% knew the name of this law. This figure is considerably low compared to those observed in Nigeria [23] and India [24], where 69% and 59,7% of physicians declare that they had read the code of medical ethics of their countries. This discordance can be explained by the older age of the Medical Ethics Code in these two countries [25, 26] compared to that of Morocco.

Unfortunately, 90% of physicians did not receive training in research ethics during their medical curricula, despite its integration in 2015. This low prevalence could be explained by the late application and a disparity of integration of medical research ethics between faculties. This situation is not unique to Morocco but is prevalent in most developing countries, whether Arab [27, 28] or sub-Saharan African countries. A study that included 42 such countries found that only 36% of all institutions surveyed provided ethics training to their staff conducting research in the field of health [29]. This is unacceptable and underscores the importance of integrating research ethics as a compulsory subject in the medical curriculum, especially since it has been shown to have a positive impact on all aspects of healthcare and research [30]. The WMA has recommended that medical research ethics should be included in undergraduate medical curricula, with a sufficient number of qualified teachers [31].

The low knowledge score observed in this study is consistent with the results of studies conducted in Sri Lanka and India, where low levels of ethical knowledge were reported among physicians (18.8% and 30% successively) [24, 32]. However, the high attitude score among physicians of this study is consistent with the results of a Jordanian study that found good attitudes among resident physicians, especially regarding consent and confidentiality [17].

In this study, it was found that physicians who had received training in research ethics during their medical curriculum had statistically higher knowledge and attitudes scores. The importance of integrating research ethics into the medical curriculum was confirmed by two studies conducted in Nigeria, which demonstrated that such training improved researchers’ knowledge [33, 34]. However, a more recent study from the same country found no association between ethics training and the level of knowledge of its researchers and which explained this observed difference by the difference in training content or methodologies used by the trainers [16]. Therefore, continuous training in medical research ethics is essential for improving the attitudes of doctors, as demonstrated by a Sri Lankan study, where 95.3% of participating doctors identified the need for continuous training in medical ethics to improve their knowledge and consequently their attitudes towards research work [32]. The same study also revealed that 79.2% of its participating doctors believe that junior doctors tend to follow the attitudes of their seniors, highlighting the importance of the suitable model to follow, that it positively influences doctors in training to instill in them good medical ethics practices.

This study also found that the participation of physicians in scientific research is insufficient (12.9%), which is the case for most developing countries, where the quantity and quality of scientific research remains considerably weak [35], despite that research on human subjects continues to increase in these countries [36, 37]. This low quality of studies has a significant impact on publications, with only 2% of scientific publications in indexed journals originating from developing countries [38]. Adequate practices of the participating physicians were associated with having already heard of medical research ethics, which is consistent with the data of the study having shown that training and institutional development are the key elements in strengthening research capacities in developing countries [39].

This study has several strengths. Firstly, it is the first to evaluate knowledge, attitudes, and practices related to research ethics among Moroccan interns and residents, and to investigate the factors influencing each of these three evaluated components. Secondly, this is a multicenter study that included a large sample of doctors practicing in various universities and regional hospitals in Morocco. Therefore, its results can be extrapolated to all Moroccan doctors. Moreover, the study used a valid questionnaire with good reliability, as well as convergent and divergent validity. Additionally, multivariate statistical analysis was used to account for various confounding factors. The main limitations of this study are associated with potential biases inherent in electronic studies, such as self-selection and the influence of social media participation. Additionally, the limited number of doctors who had previously participated in medical research studies posed a challenge, restricting our ability to thoroughly examine the influence of knowledge and attitudes on participants’ practices.

## Conclusion

Despite Morocco’ efforts in research ethics, such as the promulgation of law 28 − 13 and the integration of research ethics into the medical curriculum, the knowledge, attitudes and practices of Moroccan physicians in this area remain weak. Therefore, reinforcing efforts to improve these components is essential to increase the quantity and quality of research works conducted in the country.

### Electronic supplementary material

Below is the link to the electronic supplementary material.

Supplementary Material 1

## Data Availability

The datasets used and analyzed during the current study are available from the corresponding author on reasonable request.
