# Supplementary material for: Knowledge, attitudes, and practices of the ethics in medical research among Moroccan interns and resident physicians
Source: BMC Med Ethics. 2024 Mar 20;25:33. doi: 10.1186/s12910-024-01029-9 (PMC10953237; doi:10.1186/s12910-024-01029-9)
Supplement: Supplementary file 1 — Supplementary Material 1 [file 12910_2024_1029_MOESM1_ESM.pdf]

Scale "Knowledge, attitudes, and practices of the ethics in medical research among Moroccan interns and resident physicians"

Questions

I. Knowledge Related to Biomedical Research Ethics

1. ***How do you define biomedical research ethics?***
☐ Set of rules to be followed for conducting research
☐ Set of rules for the protection of research participants
☐ Obtaining informed consent from subjects before any research begins
☐ Respecting individuals participating in research
☐ I don't know
☐ Other, specify……………

2. Are you aware of any laws governing research ethics in Morocco?
☐ Yes ☐ No ☐ I don't know

3. If yes, which one(s)?
☐ Law n° 28-13
☐ Law n° 16-98
☐Law n° 03-94
☐ Other, specify……………
☐ I don't remember

4. Which of the following document(s) is/are considered foundational in research ethics?
☐ Nuremberg Code
☐ Helsinki Declaration
☐ Belmont Report
☐ International ethical guidelines for biomedical research involving human subjects
☐ I don't know
☐ Other, specify……………

5. In your opinion, what are the fundamental principles of research ethics involving human subjects?
☐ Respect for autonomy
☐ Beneficence
☐ Justice
☐ Non-maleficence
☐ Consent
☐ Confidentiality
☐ Conflict of interest
☐ Other, specify……………
☐ I don't know

This questionnaire was developed by the Laboratory of Epidemiology, Clinical Research, and Community Health of the Faculty of Medicine, Pharmacy, and Dental Medicine of Fez, with the contribution of Dr. Ibtissam EL HARCH and under the supervision of Professor Karima EL RHAZI

Knowledge about Informed Consent in Biomedical Research Ethics

	Correct	*Incorrect*	*Not sure*
6. It is essential to obtain informed consent from participants who are involved in research before starting the study.			
7. Informed consent includes the autonomy of subjects so they can withdraw themselves from the study at any time.			
8. Patients should not be informed about the potential risks of a study because they may choose not to participate in the study.			

Knowledge about the Principle of Justice in Biomedical Research Ethics

	Correct	*Incorrect*	*Not sure*
9. Risks and benefits of research should be shared equally among study participants.			
10. Researchers can exclude individuals from participating in a research project based on characteristics such as culture, language, religion, race, disability, sexual orientation, ethnic origin.			
11. Researchers planning to deliberately exclude certain groups from their research project must specify the reasons for exclusion to their research ethics committees.			

Knowledge about Confidentiality in Biomedical Research Ethics

	Correct	*Incorrect*	*Not sure*
12. Confidentiality is breached if a physician/researcher discloses information that puts the participant at risk of injury/harm/illness.			
13. The use of anonymous data is not a means of protecting privacy and confidentiality.			

Knowledge about Vulnerability in Biomedical Research Ethics

	correct	*Incorrect*	*Not sure*
14. Vulnerable groups such as children and the mentally ill can give their informed consent.			
15. If no legal representative of a vulnerable person is available to give informed consent on their behalf, they can still be included in the research.			
16. The judgment that a person lacks autonomy should be regularly reassessed.			

Knowledge about Research Ethics Committees

	Yes	*No*	*I don't know*
17. Do you know if there are ethics committees in Morocco?			
18. Is there a biomedical research ethics committee at your faculty or healthcare institution?			

19. What functions are generally attributed to research ethics committees?

☐ Review the ethical aspects of research
☐ Determine if informed consent is required
☐ Determine if consent is compliant
☐ Review the scientific design of research
☐ Protect the well-being and rights of research subjects
☐ Make research more difficult to conduct
☐ I don't know
☐ Other, specify……………

This questionnaire was developed by the Laboratory of Epidemiology, Clinical Research, and Community Health of the Faculty of Medicine, Pharmacy, and Dental Medicine of Fez, with the contribution of Dr. Ibtissam EL HARCH and under the supervision of Professor Karima EL RHAZI

Attitudes Related to Biomedical Research Ethics

Attitudes Regarding Confidentiality

	Yes	*No*	*Not sure*
1. Do you think a physician/researcher should always seek permission or inform the participant before breaking confidentiality?			
2. Do you think the confidentiality of participant information should be maintained as much as possible, except in situations where there is a risk of harm to others?			
3. Do you think informed consent forms should be kept locked and separate from research records to protect the privacy and confidentiality of the participant?			

Attitudes Regarding Informed Consent

	Yes	*No*	*Not sure*
4. Do you think informed consent should be explained to the participant in their local language?			
5. Do you think patients should be informed if they will be compensated for injury due to the protocol?			
6. Do you think patients should be informed of comprehensive information about the research protocol (duration, risks, outcomes, etc.)?			
7. Do you think informed consent from patients is required when using their biological samples in research?			

Attitudes Regarding Situations of Vulnerability

	Yes	*No*	*Not sure*
8. Do you think it is prudent to conduct research on a vulnerable subject (mentally ill, pregnant woman, person deprived of liberty) when this research can be done on a non-vulnerable subject?			
9. Do you think individuals with mental illness (or other vulnerable individuals) cannot make decisions regarding their participation in a research study?			
10. Do you think there should be a legal representative during the informed consent process for individuals with mental illness (or other vulnerable individuals)?			

Attitudes Regarding Conflicts of Interest

	Yes	*No*	*Not sure*
11. Do you think it is ethical to recommend patients' participation in biomedical research if you are under any financial, administrative, hierarchical, or political incentives?			
12. In your opinion, is it acceptable to inappropriately modify or delete research results due to financial or administrative or hierarchical or political pressure?			
13. Do you think doctors are required to inform research participants of the existence of any conflicts of interest and how it is managed?			

Attitudes Regarding Research Ethics Committees and Research Ethics Education

	Yes	*No*	*Not sure*
14. Do you think research involving human subjects should be monitored by ethics committees?			

This questionnaire was developed by the Laboratory of Epidemiology, Clinical Research, and Community Health of the Faculty of Medicine, Pharmacy, and Dental Medicine of Fez, with the contribution of Dr. Ibtissam EL HARCH and under the supervision of Professor Karima EL RHAZI

II. Practices Related to Biomedical Research Ethics

Have you ever participated in health research?

☐ Yes ☐ No ☐ I don't remember

If yes, answer the questions in this section; if not, the questionnaire is finished

	Yes	***No***	***I don't remember***
1. Did you request informed consent during your research work?			
2. Did you explain to the participant that they were participating in a research-based study?			
3. Did you explain informed consent to the participant in their local language?			
4. Did you obtain informed consent from the guardian or legal representative of a vulnerable participant (child, mentally ill, disabled...)?			

This questionnaire was developed by the Laboratory of Epidemiology, Clinical Research, and Community Health of the Faculty of Medicine, Pharmacy, and Dental Medicine of Fez, with the contribution of Dr. Ibtissam EL HARCH and under the supervision of Professor Karima EL RHAZI

Answers :

The correct choices are highlighted in a different color.

I. Knowledge Related to Biomedical Research Ethics

1. ***How do you define biomedical research ethics?***

☐ Set of rules to be followed for conducting research
☐ Set of rules for the protection of research participants
☐ Obtaining informed consent from subjects before any research begins
☐ Respecting individuals participating in research
☐ I don't know
☐ Other, specify……………

2. Are you aware of any laws governing research ethics in Morocco?

☐ Yes ☐ No □ I don't know

3. If yes, which one(s)?

☐ Law n° 28-13
☐ Law n° 16-98
☐Law n° 03-94
☐ Other, specify……………
☐ I don't remember

4. Which of the following document(s) is/are considered foundational in research ethics?

☐ Nuremberg Code
☐ Helsinki Declaration
☐ Belmont Report
☐ International ethical guidelines for biomedical research involving human subjects
☐ I don't know
☐ Other, specify……………

5. In your opinion, what are the fundamental principles of research ethics involving human subjects?

☐ Respect for autonomy
☐ Beneficence
☐ Justice
☐ Non-maleficence
☐ Consent
☐ Confidentiality
☐ Conflict of interest
☐ Other, specify……………
☐ I don't know

Knowledge about Informed Consent in Biomedical Research Ethics

	Correct	*Incorrect*	*Not sure*
6. It is essential to obtain informed consent from participants who are involved in research before starting the study.	✓		
7. Informed consent includes the autonomy of subjects so they can withdraw themselves from the study at any time.	✓		
8. Patients should not be informed about the potential risks of a study because they may choose not to participate in the study.		✓	

This questionnaire was developed by the Laboratory of Epidemiology, Clinical Research, and Community Health of the Faculty of Medicine, Pharmacy, and Dental Medicine of Fez, with the contribution of Dr. Ibtissam EL HARCH and under the supervision of Professor Karima EL RHAZI

Knowledge about the Principle of Justice in Biomedical Research Ethics

	Correct	*Incorrect*	*Not sure*
9. Risks and benefits of research should be shared equally among study participants.	✓		
10. Researchers can exclude individuals from participating in a research project based on characteristics such as culture, language, religion, race, disability, sexual orientation, ethnic origin.		✓	
11. Researchers planning to deliberately exclude certain groups from their research project must specify the reasons for exclusion to their research ethics committees.	✓		

Knowledge about Confidentiality in Biomedical Research Ethics

	Correct	*Incorrect*	*Not sure*
12. Confidentiality is breached if a physician/researcher discloses information that puts the participant at risk of injury/harm/illness.	✓		
13. The use of anonymous data is not a means of protecting privacy and confidentiality.		✓	

Knowledge about Vulnerability in Biomedical Research Ethics

	Correct	*incorrect*	*Not sure*
14. Vulnerable groups such as children and the mentally ill can give their informed consent.		✓	
15. If no legal representative of a vulnerable person is available to give informed consent on their behalf, they can still be included in the research.		✓	
16. The judgment that a person lacks autonomy should be regularly reassessed.	✓		

Knowledge about Research Ethics Committees

	Yes	*No*	*I don't know*
17. Do you know if there are ethics committees in Morocco?	✓		
18. Is there a biomedical research ethics committee at your faculty or healthcare institution?	The Moroccan cities that have ethics committees for biomedical research are:<br>• Casablanca,<br>• Rabat,<br>• Marrakech,<br>• Oujda,<br>• Fes.		

19. What functions are generally attributed to research ethics committees?

☐ Review the ethical aspects of research
☐ Determine if informed consent is required
☐ Determine if consent is compliant
☐ Review the scientific design of research
☐ Protect the well-being and rights of research subjects
☐ Make research more difficult to conduct
☐ I don't know
☐ Other, specify……………

This questionnaire was developed by the Laboratory of Epidemiology, Clinical Research, and Community Health of the Faculty of Medicine, Pharmacy, and Dental Medicine of Fez, with the contribution of Dr. Ibtissam EL HARCH and under the supervision of Professor Karima EL RHAZI

Attitudes Related to Biomedical Research Ethics

Attitudes Regarding Confidentiality

	Yes	*No*	*Not sure*
1. Do you think a physician/researcher should always seek permission or inform the participant before breaking confidentiality?	✓		
2. Do you think the confidentiality of participant information should be maintained as much as possible, except in situations where there is a risk of harm to others?	✓		
3. Do you think informed consent forms should be kept locked and separate from research records to protect the privacy and confidentiality of the participant?	✓		

Attitudes Regarding Informed Consent

	Yes	*No*	*Not sure*
4. Do you think informed consent should be explained to the participant in their local language?	✓		
5. Do you think patients should be informed if they will be compensated for injury due to the protocol?	✓		
6. Do you think patients should be informed of comprehensive information about the research protocol (duration, risks, outcomes, etc.)?	✓		
7. Do you think informed consent from patients is required when using their biological samples in research?	✓		

Attitudes Regarding Situations of Vulnerability

	Yes	*No*	*Not sure*
8. Do you think it is prudent to conduct research on a vulnerable subject (mentally ill, pregnant woman, person deprived of liberty) when this research can be done on a non-vulnerable subject?		✓	
9. Do you think individuals with mental illness (or other vulnerable individuals) cannot make decisions regarding their participation in a research study?	✓		
10. Do you think there should be a legal representative during the informed consent process for individuals with mental illness (or other vulnerable individuals)?	✓		

Attitudes Regarding Conflicts of Interest

	Yes	*No*	*Not sure*
11. Do you think it is ethical to recommend patients' participation in biomedical research if you are under any financial, administrative, hierarchical, or political incentives?		✓	
12. In your opinion, is it acceptable to inappropriately modify or delete research results due to financial or administrative or hierarchical or political pressure?		✓	
13. Do you think doctors are required to inform research participants of the existence of any conflicts of interest and how it is managed?	✓		

Attitudes Regarding Research Ethics Committees and Research Ethics Education

	Yes	*No*	*Not sure*
14. Do you think research involving human subjects should be monitored by ethics committees?	✓		

This questionnaire was developed by the Laboratory of Epidemiology, Clinical Research, and Community Health of the Faculty of Medicine, Pharmacy, and Dental Medicine of Fez, with the contribution of Dr. Ibtissam EL HARCH and under the supervision of Professor Karima EL RHAZI

II. Practices Related to Biomedical Research Ethics

Have you ever participated in health research?

☐ Yes ☐ No ☐ I don't remember

If yes, answer the questions in this section; if not, the questionnaire is finished

	Yes	*No*	*I don't remember*
1. Did you request informed consent during your research work?	✓		
2. Did you explain to the participant that they were participating in a research-based study?	✓		
3. Did you explain informed consent to the participant in their local language?	✓		
4. Did you obtain informed consent from the guardian or legal representative of a vulnerable participant (child, mentally ill, disabled...)?	✓		

This questionnaire was developed by the Laboratory of Epidemiology, Clinical Research, and Community Health of the Faculty of Medicine, Pharmacy, and Dental Medicine of Fez, with the contribution of Dr. Ibtissam EL HARCH and under the supervision of Professor Karima EL RHAZI

Manual for Calculating Scores

Three scores are created to assess the levels of knowledge, attitudes, and practices of physicians regarding the ethics of medical research.

- ✓ The knowledge section consists of 19 items related to the definition of research ethics, Moroccan legislation governing the ethics of medical research, existing foundational documents, fundamental principles of medical research ethics, and ethics committees.
- ✓ The attitudes section includes 14 items covering different attitudes adopted by physicians regarding consent, vulnerability, confidentiality, conflicts of interest, and ethics committees.
- ✓ The practices section comprises 4 items on the practices of physicians who have participated in medical research studies, focusing on the consent procedure. (**Note** : The practices score is only calculated for participants who answered **"YES"** to the question : ***Have you ever participated in health research ?).***

To calculate each of these three scores, the following values are assigned:

- ✓ 1 for each correct answer (an answer is considered correct if the participant selects all the correct options),
- ✓ 0 for each incorrect answer (an answer is considered incorrect if the participant does not select all the correct options).

The total score for each section is calculated by adding the results of the corresponding items, resulting in total scores ranging from:

- ✓ 0 to 19 for knowledge,
- ✓ 0 to 14 for attitudes,
- ✓ 0 to 4 for practices.

Each of the three scores is categorized into two levels of proficiency based on their 75th percentile. The 75th percentile acts as a threshold to distinguish between higher and lower proficiency:

- ✓ knowledge score: 75th percentile is 14.5,
  - Good knowledge is a score of 14.5 or higher
  - Poor knowledge is a score below 14.5
- ✓ Attitudes score: 75th percentile is 10.5,
  - Good attitudes are a score of 10.5 or higher
  - Poor attitudes are a score below 10.5
- ✓ practices score: 75th percentile is 3,
  - Good practices are a score of 3 or higher
  - Poor practices are a score below 3.
  - Poor practices < 3.

This questionnaire was developed by the Laboratory of Epidemiology, Clinical Research, and Community Health of the Faculty of Medicine, Pharmacy, and Dental Medicine of Fez, with the contribution of Dr. Ibtissam EL HARCH and under the supervision of Professor Karima EL RHAZI
